# Are lower urinary tract symptoms in men associated with cardiovascular diseases in a primary care population: a registry study

**DOI:** 10.1186/1471-2296-15-9

**Published:** 2014-01-14

**Authors:** Inge I Bouwman, Boudewijn J Kollen, Klaas van der Meer, Rien JM Nijman, Wouter K van der Heide

**Affiliations:** 1 Department of general practice, University of Groningen, University Medical Center Groningen, Antonius Deusinglaan 1, Groningen, AV 9713, Netherlands; 2 Department of urology, University of Groningen, University Medical Center Groningen, Hanzeplein 1, Groningen, GZ 9713, Netherlands

## Abstract

**Background:**

Although lower urinary tract symptoms (LUTS) seem to be related to cardiovascular disease (CVD) in men, it is unclear whether this relationship is unbiased. In order to investigate this relationship, we used longitudinal data for establishing the possible predictive value of LUTS for the development of CVD in a primary care population.

**Methods:**

We performed a registry study using data from the Registration Network Groningen (RNG). All data from men aged 50 years and older during the study period from 1 January 1998 up to 31 December 2008 were collected. Cox proportional hazard regression analysis was used to determine the association between the proportions of CVD (outcome) and LUTS in our population.

**Results:**

Data from 6614 men were analysed. The prevalence of LUTS increased from 92/1000 personyears (py) in 1998 up to 183/1000 py in 2008. For cardiovascular diseases the prevalence increased from 176/1000 py in 1998 up to 340/1000 py in 2008. The incidence numbers were resp. 10.2/1000 py (1998) and 5.1/1000 py (2008) for LUTS, and 12.9/1000 py (1998) and 10.4/1000 py (2008) for CVD. Of all men, 23.2% reported CVD (41.1% in men with LUTS vs 19.5% in men without LUTS, p < 0.01). The hazard ratio of LUTS for cardiovascular events, compared to no LUTS, in the adjusted multivariate model, was 0.921(95% CI: 0.824 - 1.030; p = 0.150).

**Conclusion:**

Based on the results, LUTS is not a factor that must be taken into account for the early detection of CVD in primary care.

## Background

Cardiovascular diseases (CVD) are a major cause of morbidity and mortality. Worldwide, CVD is responsible for an estimated 35 million deaths each year [1,2].

The recently published guidelines ‘Prevention of cardiometabolic diseases’ [3] urge general practitioners (GPs) to play a proactive role in secondary prevention of their patients at risk, and to manage subsequent intervention [3,4]. Identifying associated morbidities that may precede CVD could assist the GP in this role.

Research in the past decade has revealed an association between CVD and erectile dysfunction (ED) in community based and clinical studies [5-7]. The incidence of coronary heart diseases in Dutch men, in 2007, increases from 5/1000 men aged 50 up to 30/1000 men aged 80 [3]. The incidence of ED in general practice is, however, low (1.7/1000 men/year) [8]; using ED to help identify patients at risk for CVDs will not improve the efficacy of prevention activities. On the other hand ED is also associated with Lower Urinary Tract Symptoms (LUTS) in both community and clinically based populations [9-13]. The incidence of LUTS in the general practice population increases with age, from 2.2 in men aged < 45 years up to 18.7/1000 patients/year for men aged 85 years and older [14-16]. For the GP, it is therefore a more useful instrument in case finding of individuals with CVD than ED (incidence in primary care population: 1.7/1000 patients/year, increasing up to 5.6/1000 patients/year for men aged 65–74 years [8]). A few clinical studies have reported a cross-sectional relationship between LUTS and CVD [17-26]. Also, in one community based longitudinal study, a longitudinal relationship between LUTS and CVD was found [25]. However, the relationship between LUTS and CVD has not yet been demonstrated in a primary care setting.

LUTS and CVD share some risk factors such as obesity, diabetes, hypertension, smoking, and ageing [23,24]. The underlying pathophysiological relationship could be explained by fluid shifts, hormonal and autonomic nervous disturbances caused by hypertension and heart failure, but also by waking due to nycturia [17-26]. Endothelial dysfunction in the pelvic vascular system might contribute to bladder dysfunction with rising age [5]. Also, diabetes mellitus can lead to LUTS via neurogenic bladder dysfunction with detrusor underactivity [5].

The hypothesis that LUTS could be associated with the development of CVD still needs to be confirmed in primary care. The only evidence to date is one longitudinal, community-based study conducted on a small group of men with severe LUTS [25]. Therefore, the objective of our study is to explore the relationship between LUTS and CVD in a primary care population.

## Methods

We performed a registry study using data from the Registration Network Groningen (RNG), one of several registration networks in the Netherlands. These registration networks carry out research on data derived from the electronic registration of daily patient care in their participating general practices. The Registration Network Groningen was established in 1989, and has three practices in the north of the Netherlands, with an annual population of approximately 30,000 patients [27].

In the Netherlands, the GP is the gatekeeper in the Dutch health-care system controlling access to specialized medical care. Virtually all non-institutionalized Dutch citizens are registered with a GP so the total practice population represents the general population [28].

GPs working in the RNG-practices use a structured medical record, in which all patient contacts are registered. This includes reason for encounter, medical diagnosis (according to the International Classification of Primary Care (ICPC) [29]), applied treatment (among which prescriptions, using the Anatomical Therapeutic Chemical (ATC) codes [30], and referrals), but also cause of death. The database also includes population dynamics, such as date of entry and departure from the database.

From the RNG registration, we selected all data from men aged 50 years and older (age at any time during the study period: 1January 1998–31 December 2008).

Patients with a history of Prostate Cancer, as well as men with a history of CVD were excluded for longitudinal analysis. Access to the patient’s medical history was a prerequisite. We collected the following data from the registration: date of birth, date of entry in the study, date of and reason for leaving the registration, GP code, patient contacts (ICPC codes), prescriptions (ATC codes) and ICPC codes attached to these medications, and hospital referrals. From this information, we calculated age from date of birth and number of person years in study. All of the data were anonymised. We received ethical permission to access the Registration Network Groningen from the Medical Ethical Research Board, University Medical Center Groningen (M13.132482).

### Definitions

Cardiovascular event is defined as a documented acute myocardial infarction, chronic ischaemic heart disease, transient ischemic attack or stroke in the medical records of the GPs [31]. Table 1 provides the ICPC codes as registered by the RNG-GPs that were used for this study and recode this according to the ICPC. For each participant, we defined CVD-status at baseline (01-01-1998), or at date of study entry, as being present (previous cardiovascular event before inclusion in the study) or absent.

**Table 1:** Definitions of study parameters and ICPC codes

**Study parameter**	**ICPC code and definition**
**Lower urinary tract symptoms**	U02: frequency, U05: other voiding symptoms, U07: other symptoms, U13: other symptoms bladder, U29: other symptoms urinary tract, Y06: symptoms prostate, Y85: benign prostate hypertrophy
**Erectile dysfunction**	Y07: symptoms sexual potential
**Cardiovascular diseases**	K74: angina pectoris, K75: acute myocardial infarction, K76: other chronic ischaemic heart disease, K89: transient ischaemic attaque, K90: cerebrovascular accident
**Cardiovascular risk factors**	K86: hypertension without organ damage, K87: hypertension with organ damage, T82 obesity Quetelet Index > 30, T83: obesity QI < 30, P17: smoking, P15: chronic alcohol abuse, T93: dislilpydemia
**Medication**	LUTS medication: selective a-1 receptor blockers, 5a1-reductase inhibitors
	Antipsychotics
	Opiates
	Antidiabetics: oral and insulin therapy
	Antihypertensives: ACE inhibitors, AII receptor blockers, B-blockers, Calcium antagonists, diuretics
	Cholesterol reducing drugs
	Anti Impotence pills
	Parkinson medication
	Tricyclic antidepressants
	Anticoagulant drugs

Lower urinary tract symptoms (LUTS) include the sensation of not urinating completely, withholding urinating, and difficulty voiding. This may include having a stop-and-go urinary flow and getting up frequently at night to urinate [32]. There is no specific ICPC code for LUTS, therefore we defined LUTS by all relevant ICPC codes or use of LUTS medication (Table 1).

Erectile dysfunction is defined as the persistent inability to achieve or maintain an erection suficient for satisfactory sexual performance [33], in this study defined by the ICPC code Y07: symptoms sexual potential.

### Statistical analysis

Descriptive statistics were used to compare the baseline characteristics. Continuous variables are presented in means and confidence intervals, and nominal variables reported in modi. Prevalences (and incidence numbers for LUTS and CVD) for LUTS, CVD, hypertension, Erectile dysfunction, and Diabetes mellitus were calculated by means of prevalence/1000 personyears for the first and last studyyear (1998 and 2008).

Cox proportional hazard regression analysis was used to determine the association between the proportions of CVD (outcome) and LUTS in our population. We used age as time factor in a Cox proportional hazard regression analysis to determine the association between CVD (outcome) and LUTS in our population. In this open cohort, time to event (CVD) was calculated from the patient’s date of birth to event (i.e. diagnosis of LUTS) and data of subjects were censored in case leaving the cohort, death, or end of study.

Initially, an unadjusted analysis was performed. This association regression model was subsequently corrected for confounders. A covariate was considered a confounder in the event the beta coefficient of LUTS changed by 10 or more percent [34].

In this analysis, the following potential confounders [35] were tested: hypertension, diabetes mellitus, obesity, dyslipidaemia, depression, and antihypertensive (ACE-inhibitors, all antagonists, beta blockers, calcium antagonists, and diuretics), and statins.

In all analyses, multiple dummy variables were created for the analyses of categorical data. In addition, model assumptions were tested for compliance. All analyses were performed using SPSS version 16 based on a two-sided 0.05 significance level.

## Results

### Descriptives

From the database, we collected data from 6614 eligible men. Of these, 1165 (17.6%) reported LUTS during the study period. Table 2 describes the patient characteristics, for the total sample, and for men with and without LUTS. The prevalence of LUTS increased from 92/1000 personyears (py) in 1998 up to 183/1000 py in 2008. The incidence numbers of LUTS were 10.2/1000 py in 1998 and 5.1/1000 py in 2008.

**Table 2:** Population characteristics

***Characteristics***	***All men (n = 6614) (%)***	***Men without LUTS (n = 5449) (82.4%*** ***)***	***Men with LUTS (n = 1165) (17.6%*** ***)***	***p-value***
Age, mean (SD), y	56 (12)	54 (11)	65 (12)	
Report of cardiovascular disease (*1)	1539(23.2)	1060(19.5)	479(41.1)	0.000*
Report of hypertension (K86 + K87)	1751(26.5)	1283(23.5)	468(40.2)	0.000*
Report of erectile dysfunction (*2)	89(1.3)	56(1.0)	33(2.8)	0.000*
Report of diabetes mellitus (T90)	368(5.7)	286(5.2)	92(7.9)	0.000*
Report of obesity	260(3.9)	204(3.7)	56(4.8)	0.090
Report of current smoking	884(13.4)	751(13.8)	133(11.4)	0.031*
Report of alcohol abuse	254(3.8)	230(4.2)	24(2.1)	0.000*
Report of dislipydemia	1350(20.4)	1046(19.2)	304(26.1)	0.000*
Use of LUTS medication (*3)	7242(10.9)	5(0.1)	717(61.5)	0.000*
Use of DM medication (*4)	29(0.4)	22(0.4)	7(0.6)	0.355
Use of antihypertensives				
Use of aII antagonist	412(6.0)	256(4.7)	156(13)	0.000*
Use of ACE inhibitors	1278(19.3)	937(17.2)	341(29.3)	0.000*
Use of beta blockers	1787(27.0)	1327(24.4)	460(39.5)	0.000*
Use of calcium antagonists	867(13.1)	579(10.6)	288(24.7)	0.000*
Use of diuretics	1063(16.1)	732(13.4)	331(28.4)	0.000*
Use of statins	1145(17.3)	879(16.1)	266(22.8)	0.035*
Use of ED medication	389(5.9)	282(5.1)	107(9.1)	0.010*

Data are expressed as No. (%) unless otherwise noted. SD: standard deviation, y: year. *1: Cardiovascular Diseases include K74 (angina pectoris*)*, K75 (acute myocardial infarction), K76 (other chronic ischaemic heart diseases), K89 (TIA), and K90 (CVA). *2: Erectile Dysfunction includes: Y07 (symptoms sexual potential), *3: Use of medication for Lower Urinary tract symptoms, *4: Use of medication for Diabetes mellitus.

*: p-value significant (<0.05).

From all men, 1539 had cardiovascular diseases during the study period: 23.2% (95% CI 22.2-24.2%) (41.1% in men with LUTS vs 19.5% in men without LUTS, p < 0.01). The incidence numbers for CVD were 12.9/1000 py in 1998 and 10.4/1000 py in 2008.

Table 3 shows the outcome of LUTS and CVD by agegroups among the study population. Both LUTS and CVD are more prevalent in the highest age group. The prevalence of CVD increased from 176/1000 py in 1998 up to 341/1000 py in 2008. From all men 26.5% had hypertension, significantly more prevalent in the LUTS group (40.2% in men with LUTS vs. 23.5% in men without LUTS, p < 0.01). The prevalence of hypertension increased during the study period, from 206/1000 py in 1998 up to 387/1000 py in 2008. Also Erectile Dysfunction was more prevalent in the LUTS group (2.8% in men with LUTS vs. 1.0% in men without LUTS, p < 0.01). The prevalence of ED increased from 17/1000 py in 1998 up to 19/1000 py in 2008 during the period 1998–2008. Diabetes mellitus was prevalent in 5.7% of all men, the prevalence increased from 24/1000 py in 1998 up to 82/1000 py in 2008. The prevalence of DM was not significantly (p = 0.85) different in the LUTS group compared to the non-LUTS group. Dyslipidemia was prevalent in 20.4% of all patients. It was significant more prevalent in the LUTS group (p 0.005) than in the non LUTS group. The prevalences of lifestyle factors as obesity, smoking, alcohol abuse were 3.9, 13.4, and 3.8% respectively. There were no significant differences between the LUTS and non-LUTS group for lifestyle factors (p-values are 0.42, 0.52, and 0.18 respectively).

**Table 3:** CVD status by age and LUTS status among the study population

	**No LUTS, n = 5449**			**LUTS, n = 1165**				
	***N (% within presence of LUTS)***			***N (% within abcence of LUTS)***			***CVD, n*** **= 1539**	
**CVD status**	***No CVD***	***CVD***	***Subtotal no LUTS, n (%)***	***No CVD***	***CVD***	***Subtotal LUTS n (%)***	***Subtotal CVD n (%)***	**p-value**
**Age groups**								
50**–**54 yrs	3015 (90.7)	309 (9.3)	3324 (61.0)	223 (85.1)	39 (14.9)	262 (22.5)	348 (22.6)	0.003
55-59 yrs	509 (77.5)	148 (22.5)	657 (12.1)	92 (66.2)	47 (33.8)	139 (11.9)	159 (10.3)	0.005
60-64 yrs	333 (72.1)	129 (27.9)	462 (8.5)	82 (57.3)	61 (42.7)	143 (12.3)	190 (12.3)	0.001
65-69 yrs	215 (57.8)	157 (42.2)	372 (6.8)	86 (49.9)	88 (50.6)	174 (14.9)	245 (15.9)	0.067
70-78 yrs	317 (50)	317 (50)	634 (11.6)	203 (45.4)	244 (45.4)	447 (38.4)	561 (36.5)	0.137
All ages	4389 (80.5)	1060 (19.5)	5449 (100%)	686 (58.9)	479 (41.1)	1165 (100%)	1539 (100%)	0.000

LUTS: Lower Urinary Tract Symptoms, CVD: cardiovascular disease.

### Longitudinal analysis

There is a significant unadjusted univariate relationship between the proportions cardiovascular events and LUTS (HR 1.16, 95% CI: 1.035 - 1.291; p = 0.010). However, the hazard ratio of LUTS for cardiovascular events, compared to no LUTS, in the adjusted multivariate model, was non-significant (HR = 0.921, 95% CI: 0.824 - 1.030; p = 0.150). Therefore, men with LUTS were not more likely to develop CVD, at any time, than men without LUTS. In our analysis, hypertension, ACE-inhibitors, All antagonists, beta blockers, and calcium antagonists were found to confound this relationship. In this population, cardiovascular disease was not associated with LUTS (Table 4).

**Table 4:** The association between LUTS and CVD from longitudinal analyses, final model

**Outcome**	**Unadjusted**	Multivariate adjusted *
	HR (95% CI, p-value)	HR (95% CI, p-value)
CVD	1.16 (1.035 - 1.291, p 0.010)	0.921 (0.824-1.030, p 0.150)

*Variables in multivariate analysis: Hypertension, ACE inhibitors, AII antagonists, beta blockers, Calcium antagonists.

## Discussion

### Main findings

Nowadays, when a patient consults his general practitioner for LUTS, it is not customary to search for comorbidities such as CVD. However Dynamic cohort and clinical studies suggest a correlation between LUTS and CVD. To the best of our knowledge this is the first study that has been done to establish the relationship between LUTS and CVD in a primary care population. This is an important population because the GP plays an increasingly prominent role with respect to the early detection of patients at risk, for example, for cardiovascular diseases [3,4]. In this study, we investigated 1165 men with LUTS. Based on the results, LUTS is not a factor that must be taken into account for the detection of CVD, in primary care.

### Interpretation of findings in relation to previously published work

Other studies report the association between LUTS and CVD [17,18,36]. The results suggest that vascular risk factors seem to be associated with the presence and degree of LUTS, prostate size or outflow obstruction. Vascular diseases such as atherosclerosis and endothelial dysfunction in the pelvic vascular system are one of the possible mechanisms contributing to bladder dysfunction with age [17]. Increased sympathetic activity and/or a1-adrenoreceptor activity might be a common pathway for both hypertension and LUTS, and might explain the improvement in LUTS with the use of a1- adrenoreceptor antagonist [36]. Kim et al. found that men with 3 or more vascular risk factors were 3 times more likely to have moderate/severe LUTS than men without vascular risk factors (OR 3.6 (95% CI 1.19-10.62, p0.024). However, these studies are mostly clinical studies [17-22]. There are often considerable methodological and population differences between them. This latter is an important point as we cannot generalise the results from other studies (all clinical- or community based studies) to the patient population of the GP. Also the cross-sectional setting of the studies makes them not suitable to analyse causal relationships.

Recently, a community-based retrospective, cross-sectional and longitudinal study into this relationship was carried out [25]. Wehrberger et al. concluded that men with severe LUTS have an increased risk of developing CVD. They investigated a large group of people with LUTS (n = 2092). However, the conclusions of their study were based on just 1% of the total study population. Another population based longitudinal study did not found an association between LUTS and CVD, but was especially focussed on nycturia as a subcategory of LUTS [37].

### Strengths and limitations

The strength of our study is the longitudinal analysis, and the large cohort of 6615 men. Using data from electronic medical records from general practices makes it possible to compare the prevalence of CVD between different patient groups in the same study population. We also had information about the sequence of the diseases, LUTS and CVD events, and hence a possible relationship between LUTS and subsequent CVD could be taken into the statistical analyses.

According to previous articles about RNG data, this study population is representative for the Dutch population [38].

A limitation of this study is having access to large patient populations in a health registry with data that are not collected in a structural way. Patients are not measured periodically. Important confounders, such as dyslipidemia, smoking and obesity, are not always registered in the electronical medical records of GPs. For example, in this study dyslipidemia is more frequent in men with LUTS. However, the prevalence of dyslipidemia in all subjects is lower than the Dutch prevalence numbers [39]. Because we cannot exclude underregistration of cardiovascular risk factors, we chose not to draw any conclusions from this finding. An additional investigation into medical files could yield some of the unknown information about cardiovascular risk factors, in a longitudinal study-setting.

Because of the absence of an ICPC code for LUTS, we assumed the presence of LUTS if one or more of these ICPC codes were identified (Table 1). Unfortunately, this did not always agree with the LUTS definition from other studies. As we did not measure the International Prostate Symptom Score (IPSS) of men with LUTS, we were unable differentiate between men with mild, moderate or severe LUTS.

Another factor that should be considered is the underreporting of LUTS, the so-called iceberg phenomenon: [15,40,41] i.e. the reported incidence and population prevalence differ considerably (contact with GP for LUTS: from 4-9% up to 23%) [15,42]. Consequently, fewer LUTS cases are identified by the GP than are actually present in the practice population. The lack of relationship established between LUTS and CVD might therefore be incorrect.

## Conclusions

To the best of our knowledge, this is the first study that investigated the relationship between LUTS and cardiovascular diseases in primary care. It suggests that a GP does not have to complete a full cardiac risk profile in men who present with LUTS symptoms for the first time.

## Abbreviations

LUTS: Lower urinary tract symptoms; CVD: Cardiovascular disease; RNG: Registration network groningen; GP: General practitioner; ED: Erectile dysfunction; ICPC: International classification of primary care; ATC: Anatomical therapeutic chemical; PY: Personyears; CI: Confidential interval; HR: Hazard ratio; OR: Odds ratio.

## Competing interests

The authors declare that they have no competing interests.

## Authors’ contributions

IIB: carried out the analysis and interpretation of data, and drafted the manuscript. BJK caried out the analysis and performed the statistical analysis, and helped to draft the manuscript. WK, KM, and JMN participated in the study design and coordination. WK helped to draft the manuscript and made contributions to conception and design of the study. All authors read and approved the final manuscript.

## Pre-publication history

The pre-publication history for this paper can be accessed here:

http://www.biomedcentral.com/1471-2296/15/9/prepub
